# Psychosocial demands and resources for working time organization in GP practices. Results from a team-based ethnographic study in Germany

**DOI:** 10.1186/s12995-021-00336-w

**Published:** 2021-10-18

**Authors:** Christine Preiser, Elena Tsarouha, Birgitta Weltermann, Florian Junne, Tanja Seifried-Dübon, Sigrid Hartmann, Markus Bleckwenn, Monika A. Rieger, Esther Rind, on behalf of the IMPROVEjob-Consortium

**Affiliations:** 1 Institute of Occupational and Social Medicine and Health Services Research, Faculty of Medicine, University Hospital Tuebingen, Wilhelmstr. 27, 72074 Tuebingen, Germany; 2 Centre for Public Health and Health Services Research, Faculty of Medicine, University Hospital Tuebingen, Osianderstr. 5, 72076 Tuebingen, Germany; 3 Institute of Family Medicine and General Practice, Faculty of Medicine, University Hospital Bonn, Venusberg-Campus 1, 53127 Bonn, Germany; 4 Department of Psychosomatic Medicine and Psychotherapy, University Hospital Tuebingen, Osianderstr. 5, 72076 Tuebingen, Germany; 5 Department of General Practice, Medical Faculty, University of Leipzig, Ph.-Rosenthal-Str. 55, 04103 Leipzig, Germany

**Keywords:** Occupational health, Work-related psychosocial factors, Work-related stress, Primary care practice teams, General practice, Grounded theory

## Abstract

**Background:**

General practitioners (GPs) are challenged, e.g. by long working hours, and as employers they are responsible for the creation of working conditions that prevent work-related psychosocial risks. Leadership behaviour plays an important role within the working conditions of employees, thus we focused on two research questions: To what extent and how do GPs fulfil their role as entrepreneurs and leaders responsible for occupational safety and health of the team members in the organization of working time of the employees? What psychosocial factors result from the way of organization of working time for the practice team?

**Methods:**

Data was collected by participant observations, individual interviews with six GPs, and five focus group discussions with 19 members of the practice staff in total. We gained access to five general practices through a teaching network associated with the Institute for General Medicine, University Hospital Essen (Germany). The analysis was carried out according to the Grounded Theory approach.

**Results:**

GPs have several roles and related tasks to fulfil in the organization of working time. This can lead to perceived psychological stress. With regard to the organization of predictable working hours, vacations and sickness absence, the GPs determined the scope of action of the practice assistants. The delegation of these tasks took place to varying degrees and resulted in different work-related resources and stressors.

**Conclusion:**

We described transactional and transformational leadership behaviours which are all related to specific psychosocial demands and resources and may overlap on site. Leadership training seems recommendable as part of the training of GPs and other future leaders of micro-enterprises to promote self-reflection by the entrepreneurs and leaders and strengthen occupational health of leaders and staff.

**Supplementary Information:**

The online version contains supplementary material available at 10.1186/s12995-021-00336-w.

## Background

Good working time organization is known to reduce work-related health risks and thus is an important issue of occupational health and safety [1]. The organization of work and working times comprise several dimensions, such as the duration, location, and distribution of working time as well as the intensity of work [1, 2]. Employers are responsible for creating sustainable and health-promoting working conditions [3, 4]. In general practices in Germany, general practitioners (GPs) are entrepreneurs of small or rather micro enterprises, employers, leaders and healthcare professionals at the same time. As employers, they are responsible for all aspects of occupational health and safety of their practice staff including working conditions, but may delegate these tasks to personnel. During medical studies and postgraduate medical training, GPs in Germany are usually well-trained in all aspects of the diverse roles of physicians as described e.g. by the CanMEDS framework [4, 5] adapted to the German situation [6]. Nevertheless, they have to continuously extent their skills regarding all of these roles and tasks, also including their skills as entrepreneurs and leaders of small and medium-sized enterprises (SMEs) [7].

Working time is an issue covered by legislation to protect employees based on European regulations. In Germany, legislation regulating working time and breaks policy was adopted in 1994, and the German Working Time Act provides a framework for flexible working time [8, 9]. Different regulations apply for certain professional groups e.g. for head physicians [9]. With regard to consultation hours, a framework regulates the weekly consultation hours of GPs for patients in the statutory health insurance who account for more than 85% of patients in Germany [10]. Thus, although being self-employed as practice owners, GPs as employers cannot decide completely independently on the organization of their own and staff working time. Nevertheless, they have some scope of action with regard to the implementation of the legal requirements, e.g. by offering various working time models (part-time, individual time schedules, etc.) for employees [1]. Besides legal regulations, the shortage of GPs and the ageing German population imply that a decreasing number of GPs have to care for a growing proportion of the population requiring extensive support [11]. In addition, there is a shortage of trained practice assistants (PrAs) [12], which means that GPs have difficulties to hire a sufficient number of PrAs for the given demand of medical care [13].

How working time is organized has an impact on the health and well-being of employees [2, 14, 15]. Possible critical working time attributes are, for example, alternating shifts or long working time, extensive overtime, insufficient breaks and on-call duties [3]. The physical and mental health effects of long working hours, a high variability of working hours and a lack of recovery time are well known [2, 14, 15]. However, the duration of working time alone does not in itself constitute a psychosocial demand, at least as long as the duration does not exceed approximately 45–54 h / week [16]. In Germany, GPs currently work an average of 52 h per week with a wide variation [17]. As described in the demand-control-model [18], even long working hours in combination with opportunities for development and a high scope of action may have a positive effect on a person’s health and well-being. However, long working hours combined with inadequate physical and psychosocial working conditions can pose a risk to employees’ health [19]. Thus, work-related psychosocial demands [20] can have both, positive and negative effects on health, depending on other factors such as further individual or work-related demands and individual or job-related resources [21].

In Germany, GPs and PrAs have reported high levels of perceived work-related chronic stress [22], and GPs were shown to have a high burnout prevalence [23]. Nevertheless, there are few studies investigating work-related stress and job satisfaction of GPs and practice teams [24–27]. To better understand work-related psychosocial demands and resources, we previously investigated how work is organised within general practice teams and which resources and demands are specific to the primary care setting [28, 29]. In this paper, we acknowledge that leadership behaviour plays an important role for the working conditions of employees. Our understanding of leadership follows the CanMEDS [5] framework which includes issues of management and administration also as tasks of leadership. Drawing on data from the same ethnographic study in GP practices as our prior analysis, we address the issue of working time organization focussing on two questions of interest:

- How do GPs fulfil their role as entrepreneurs and leaders responsible for the occupational safety and health of their employees regarding the organization of working time?
- What psychosocial demands and resources result from the way how working times for practice teams are organized?

## Methods

The methodological approach has been described in detail in the research protocol of this study [28]. In line with the SRQR-Criteria [30], the design of the study conforms to the guidelines for qualitative papers (supplementary material 1).

### Study design

The study applied a team-based ethnographic design [31] including participant observation and interviews with the practice owners as well as focus group discussions with staff members in five general practices in North-Rhine-Westphalia. Through the participant observations, daily work routine and respective psychosocial factors were explored. The interviews with the GPs focused on the experiences and assessments on leadership issues, patient care and team-based cooperation. The focus group discussions provided a collective view of work processes by the practice staff. By triangulating the methods and data [32], work contexts, working conditions and processes as well as the values and experiences of the practice teams were researched.

### Study population

We gained access to five general practices through a general practice network associated with the Institute of General Medicine, University Hospital Essen (Germany), a partner of the research collaboration IMPROVE*job* [33]. Selective sampling [34] was used to recruit all practice teams, considering a variety of practice characteristics to represent different practice types [in detail described by 28]. For reasons of confidentiality, no personal information of the practice teams was collected (e.g. age, gender of the employees, duration of employment). For the same reason, we decided to present the sample not along the cases, but along relevant characteristics (see Table 1).

**Table 1:** Characteristics of the study population

Characteristics	Specifications
Practice type	Single and group practices
Practice owners	Female and male between 40 and 60+ years
Location of the practice	3 practices in urban and 2 practices in rural areas
Practice staff	Mostly female, representing the age range of occupational life, higher proportion of persons of colour compared to practice owners
Number of physicians in practices	1 to 6
Number of practice staff members	5 to 29

### Data collection

To guide the participant observation, a framework was developed based on recommendations for implementing psychosocial risks assessments, including the work patterns” work content and task”, “organization of work”, “social relations”, “working environment” and “new forms of work” [3]. This provided a point of reference regarding specific work processes and work-related stress in general practices, including work organization and work time. Practice staff and patients were informed about the participant observation. The practice owners and the practice staff who took part in the interviews and the focus group discussions signed individual declarations of consent. Each participant had the possibility to revoke their consent at any time over the course of the study. All observers (ET, SH, ER) signed declarations of confidentiality.

The researchers conducting the observations had different professional backgrounds (nursing, health services research and sociology). As none of them had previously worked in general practices, the observers attended a two-day workshop developed and conducted by BW to familiarise themselves with the primary care setting. The observations took place over the course of a working week per practice. Two out of three researchers conducted the participant observation alternatingly for 2 to 4 h per day. Since the study focused on the general practice teams, no patient-related data was collected. ET, SH and ER also interviewed six GPs and conducted five focus group discussions with in total 19 members of the practice staff during the respective week of the participant observations. Using semi-structured interview guides [35], GPs and practice staff were interviewed separately in order to prevent mutual influence. All interviews and focus group discussions were audio recorded.

Data collection commenced in February 2018 and was carried out in a reciprocal process with the data analysis. As no further conceptual insights occurred during the analysis of data from the fifths practice, we concluded that data saturation was reached and data collection was finished in May 2018 [36].

### Data analysis

The recorded data was transcribed according to a simplified system [37] by a professional company. The researchers conducting the fieldwork did quality checks, de-personalization and pseudonymisation of all data. The analysis software MAXQDA 2018 [38] was used to organize the analysis.

The researchers conducting the fieldwork were responsible for data analysis following the steps of a Grounded Theory approach including open, axial and selective coding [39]. They were supported by an interdisciplinary team of researchers of the IMPROVE*job* collaboration, holding expertise in general medicine (BW, MB), psychosomatic medicine and psychology (FJ, TSD), occupational medicine (MAR) and sociology and health services research (CP). All authors were involved in the coding process to discuss the emerged core concepts and the conceptual integration of the results. A workshop with additional members of the research support group (three GPs and two PrAs) [28] ensured further credibility and transferability of the findings presented in this study.

## Results

We will give an overview over different models describing how GPs organized consultation hours. Then we will present how overall working time is organized in GP practices and how specific aspects such as breaks, sick leave and vacations are organised. All results will be illustrated by quotes from the observation protocols, interviews and focus group discussions (Tables 2, 3, 4 and 5). The selected quotes were linguistically revised and then translated from German into English by the authors. As we focused on the overall content of the data collected, no significant loss of meaning was expected due to the translation.

**Table 2:** Organization of consultation hours

Topics	Quote number	Quote
Long consultation hours	Quote 1	“We are open on [three days per week] non-stop from 8 a.m. to 6 p.m. That is 30 h already. And on two other days we are open from 8 a.m. to 1 p.m. That is another ten hours, so that is 40 h in total. And you have seen: the practice door always opens at 7:30 a.m. If you add the 2 ½ hours, the opening hours are 42 ½ hours. […] So we are open 42 ½ hours, and that is why [the physicians] have divided the working time accordingly, because nobody wants to be here for the whole 42 ½ hours.” (Interview with GP, group practice 3)
High frequency of patients	Quote 2	“Patients are narrowly clocked during the so-called acute consultation hour. The staff sways busily between the rooms. The concerns of the patients are oftentimes the same and it feels to me like a continuous loop. The computer and the list of the patients predetermine the time and cause stress, especially shortly before the transition to the appointment consultation hour. PrA 4 explains to me, that sonography appointments have to be kept on the dot, ‘or it will cause trouble.” (observation protocol, group practice 1)

**Table 3:** Organizing overall working time

Topics	Quote number	Quote
Different roles and tasks	Quote 3	“I think probably it is the coordination of everything. You are not a physician […] from 3 to 4 p.m., but you are everything at the same time. And I think that is probably it. We are not made for multitasking after all, right? Then one of [the employees] comes and says […] the printer doesn’t work anymore and the other one says the telephone system [is faulty.] I think it is the expectation that something can happen at any time which makes the day slip out of line.” (Interview with GP, group practice 5)
Decision-making authority	Quote 4	“We will have another emergency staffing this afternoon. […] This morning the team did not communicate properly at the times when it was possible […][.] Instead I [GP] was asked, in the bustle, where I was just doctor, and I said: ‘I will decide later.’ And then they decided something without consulting me again. That is a no-go.” (Interview with GP, group practice 2)
Administrative tasks	Quote 5	“Many organizational things, dictating letters, medical reports, and inquiries. All this is not achievable in the daily routine; it all takes place at the weekend or in the evening.” (Interview with GP, group practice 1)
Extended working week	Quote 6	“Yes, we certainly do not have a five-day week, but rather a six-day week. I certainly have a seven-day week at the moment, because I’m doing a little bit more. The partner physician of the practice has at least a five and a half-day week. So, work is done on half of the Saturday or half of the Sunday.” (Interview with GP, group practice 1)
Predictability of working time	Quote 7	“Predictability. As you have seen, there are […] staff members here, […] who are now back from parental leave with one or two children. They must be able to plan [their working day]. They have to know: at a certain time I can pick up my son from the childcare center. [If] the practice is so disorganized and they don’t know when they are off work, then [they] could not work here, or they would feel pressure all the time.” (Interview with GP, group practice 3)
Scope for action of the PrAs	Quote 8	“At 6:00 p.m., we are definitely done. But it is also the staff who ensure that […]. They have a little bit of control themselves, and they know a lot of the patients. Usually they know [which patients] need a lot of time. […] [The PrAs] do not arrange appointments with [these patients] at 5:45 p.m. That is self-protection […]. I think that is reasonable and understandable. I think, it is important that [the PrAs] have their own tasks and that [the GPs] don’t constantly meddle with these.” (Interview with GP, group practice 3)
Insufficient breaks	Quote 9	“I think you could relieve a lot of stress and at least have lunch on time. That is what I find most exhausting, that sometimes you are not able to take a break on time and then you work through the whole day. You may have just taken a quarter of an hour’s [break] and the work [continues] until 6, 7, 8 p.m.” (Focus group discussion among practice staff, group practice 2)
Work-privacy-balance	Quote 10	“We as mothers are finishing work quite punctually, because we have to pick up the children. But for example, I am currently working in the afternoon, […], and the end [of the working day] is just open for us, too.” (Focus group discussion among practice staff, group practice 2)

**Table 4:** Organizing breaks in daily routines

Topic	Quote number	Quote
Attitude towards the regular implementation of breaks	Quote 11	“But there is no 10:00 a.m. break. That depends on how much work needs to be done. If there is not much to do like today, then no one here will blame anyone for sitting in the kitchen. That is for sure.” (Interview with GP, group practice 3)
Absence of short breaks at certain work stations	Quote 12	“[Standing near the registration desk for about 3 h, the observer] could not observe that one of the PrAs went to the toilet or got herself something to drink. There are also no cups or glasses at the workstations.” (Observation protocol, group practice 3)
Design of break rooms	Quote 13	“From the laboratory a further room is accessible and [a] PrA presented this room to [the observer] as a break room area. There is a large dining table with four chairs. There are various cupboards and shelves along the walls. On a smaller shelf there is a coffee machine and there are several cups and glasses as well as kitchen utensils and supplies for the preparation of coffee. To the left there is a waist-high drawer cabinet with a printer, a mobile phone station and an answering machine.” (Observation protocol, single practice 1)
Social relationships	Quote 14	“So first of all the staff room is very important, where you can retreat and which is also used in the morning, because some members of the practice team have breakfast together and drink coffee before work.” (Interview with GP, group practice 5)
Absence of breaks for GPs	Quote 15	“Especially the doctors. […] Our doctors sometimes have zero rest at all. They sit here with their food and work while they eat. So they have really no rest.” (Focus group discussion among practice staff, group practice 2)

**Table 5:** Organizing predictable and unpredictable absence of staff

Topics	Quote number	Quote
Delegation	Quote 16	“The PrAs coordinate vacations among themselves. The doctor does not want to [get involved]. All [the GP] cares about is that somebody is there and the place is up and running.” (Observation protocol, group practice 5)
Adjusted patient volume	Quote 17	“We have a week of Pentecost holidays this [year]. And we don’t work during this time, because I [the GP] am not here either. The practice is closed. And afterwards [one PrA] goes on vacation for another two weeks in June because she has so much planned […]. In this case we will make fewer appointments or postpone [some]. We will manage that.” (Interview with GP, single practice 1)
Mutual social support	Quote 18	“I1: We ask each other: Can I go on vacation during this time, can we somehow organize that someone comes additionally or anything else? Sure, we... I2: And it always works out. I1: Well, I don’t think anyone would ever say no. I2: That is how it works. I1: Sure, because we respect each other’s vacation requests. I2: Anyone’s.” (Focus group discussion among practice staff, single practice 1)
Sickness absence of GPs	Quote 19	“I think my colleague and I have the same work spirit. It must happen a lot not to show up for work. This happened to me once, three years ago. I got so dizzy, I could not get up. I definitely could not work. I think in the last 27 years I have missed this one week. Apart from that, I was always here.” (Interview with GP, group practice 5)
Leadership tasks	Quote 20	“And if someone is ill, it is discussed in the morning whether to change the areas. I think it works quite well. […] But because [the doctor] is the one who is usually called if a PrA does not come. Thus, [the doctor] is the first to know when there is an absence […]. [The doctor] knows exactly which PrA is on vacation and which one is here […]. […] So when I [one PrA] arrive at 8:00 a.m., it is all arranged.” (Focus group discussion among practice staff, group practice 3)
PrAs organize sickness absence	Quote 21	“I [GP] organize absences due to illness almost never. The girls do it themselves. [They organize] who comes instead or who stays longer, […] or we cancel appointments. […] Sometimes that is just the way it is. But it must be said that they are rarely sick.” (Interview with GP, single practice 1)
PrAs have the required knowledge	Quote 22	“I1: I [PrA] think we have become pretty good at reorganizing in case of sickness absence. […] What is the boss supposed to say? I2: Exactly. I3: We have to share the work in the front. […] I2: [The doctors] have a lot of work to do. And it is actually the job of the PrAs to make house calls. I think it is pretty important that we can assess how many patients are still waiting, what other tasks need to be done and then divide that up.” (Focus group discussion among practice staff, group practice 2)
Planning of staffing ratio	Quote 23	“And since I am the personnel manager of this company, we have simply changed the number of staff, so that we do not get into shortages too easily.” (Interview with GP, group practice 2)
On-call roster	Quote 24	“I1: Due to the fact that stress has come up so often, we or [name of a certain employee] have made an on-call roster […] for the whole year. I2: Yes. That means that every week there is someone else who should theoretically have to fill in when someone else is absent. I3: So that you know right from the start: It is my turn a certain week of the year. […] This enables mothers […] to ensure childcare in order to be able to fill in flexibly.” (Focus group discussion among practice staff, group practice 1)

I1, I2: interview partner 1, interview partner 2

### Organization of consultation hours

The working hours of the GPs and the PrAs both comprised working time during the opening hours of the practice and working time behind closed practice doors. GPs in single and in group practices alike had long consultations hours*,* but it remained unclear whether there was a difference between the number of patients treated per GP in single practices compared to group practices. In group practices, physicians organised their overall working hours and their preferred consultation hours among themselves in order to ensure long practice opening hours without necessarily stretching their individual working hours [Table 2, Quote 1].

The practices observed used two different models to organize consultation hours: appointment scheduling and walk-in consultation hours. Appointment systems are characterised by patients booking specific appointments in advance and being treated in the order of appointments organized by the PrAs. Walk-in consultation hours are characterised by patients coming spontaneously to the practice and either waiting or coming back later that day to be seen by the GPs. The respective approach was usually determined by the GPs and carried out by the PrAs working at the registration desk. Several practices used hybrid models, e.g. when most parts of the GPs consultation hours were structured along appointments and a specific time frame per day was reserved to treat walk-in patients, or when most parts of the GPs consultation hours were organized as a walk-in system, but specific patients received appointments. In the hybrid models, some GPs had a higher frequency of patients during the walk-in hours, meaning more patient contacts and less time per patient [Table 2, Quote 2]. Furthermore, neither the PrAs nor the GPs would know in advance how many patients would come by and for which reasons.

Appointment scheduling reduced the number of patient contacts; hence, GPs had more time per patient, especially for those with complex diseases and situations, and the possibility to plan the order of cases (e.g. not 5 intense cases in a row). In both models, patients were seen as a ‘plannable unplannable factor’. Practice staff and GPs saw walk-in systems as a means to deal with unplannable patients in the sense of patients who became spontaneously sick or patients who were assumed to or had proven to not hold appointments. Appointment systems on the other hand offered more predictability, but still it was not certain whether additional patients would eventually show up during consultation hours.

### Organizing overall working time

GPs saw the ‘unplannable’ as an integral part of their daily routine during consultation hours [Table 3, Quote 3]. They reported that they had to carry out different tasks and roles simultaneously during this time of their working day. For example, when a spontaneous need for action emerged, such as practice staff sickness notifications or technical disruptions, GPs had to switch between different roles during consultation hours which could lead to perceived psychological stress for the GPs and within the practice team, e.g. when the GP’s self-perceived situational role was “just doctor” while the PrAs needed another situational role [Table 3, Quote 4]. The GPs regarded administrative tasks such as the writing of expert reports or accounting as impossible to be carried out during practice opening hours. For this reason, they planned these tasks to be accomplished outside the opening/consultation hours, i.e. mostly in the evening or sometimes during the weekend. This was rather phrased as additional working hours than as part of the regular working hours [Table 3, Quote 5]. Altogether, GPs highlighted their extended working week and expressed a high workload and few rest periods [Table 3, Quote 6].

In contrast to the diverse tasks of GPs besides consultation hours, PrAs usually had no work-related tasks to manage after leaving the practice. It was reported that PrAs would be contacted occasionally after working hours for practice-related questions (e.g. management of deliveries, administrative tasks). The PrAs expressed satisfaction with the flexibility of different working time models and the GPs’ overall willingness to adjust working time models and hours to the needs of the PrAs. This was discussed in the interviews and focus groups mostly in the context of compatibility of work and (child) care. Yet, when it came to day-to-day organization of working time, our observations and the interviews/focus groups showed that the organization of daily finishing time was the other side of the coin. One GP addressed three available options: (1) the GP makes sure that the finishing times match the needs of the PrAs, (2) the GP looks for PrAs who match the finishing times of the practice, (3) the finishing times and the needs of the PrAs collide and the PrAs are exposed to pressure [Table 3, Quote 7].

Some GPs saw appointment systems as a means to ensure predictability through plannable finishing times. As the organization of the appointments was delegated to the PrAs, this was meant to also provide the latter with a scope for action [Table 3, Quote 8]. In contrast, the walk-in hours were difficult to plan. Here, the immediate treatment of patients was favoured over predictable working times for PrAs and the GPs themselves. As a result, the working times could become barely plannable, which was problematized by PrAs with regards to taking (lunch) breaks and finishing time [Table 3, Quote 9 and 10]. Solutions were sought partly in the workplace when GPs and PrAs planned rosters in a way that employees with children could leave on time or at least earlier. Other solutions had to be sought by the respective PrAs in their private lives, e.g. when private plans were rescheduled spontaneously or private networks helped out with childcare.

### Organizing breaks in daily routines

Breaks are required by law to provide rest and recovery. It is the GP’s task as employer to enable breaks in terms of spatial as well as temporal dimensions. Paridon et al. [40] distinguish between micro-breaks (< 1 min), mini breaks (1-5 min), short breaks (5-10 min) and longer breaks (> 10 min). The GPs considered the practice staff self-responsible to organize at least most of their breaks if not all of them [Table 4, Quote 11]. We observed absence of micro and mini breaks at workplaces with high patient contacts, such as the registration desk. There, the PrAs rarely took micro breaks, e.g. for drinking, or mini breaks, e.g. for using the toilet [Table 4, Quote 12]. The PrAs connected this not only to a high workload, but also to the workplace design, as they were exposed to the gazes of patients at the desks and felt the urge to be visibly at work. We observed in several practices that GPs and PrAs made use of break rooms as a place for short breaks and longer breaks. The design of break rooms ranged from a small tea kitchen for one person to larger rooms with facilities for several people. The larger rooms usually held several functions: They were break rooms, but also had either a workstation integrated, or were used as storage space for materials [Table 4, Quote 13]. One GP pointed out that the break rooms were not only a room for individual recreation but also for maintaining social relationships among colleagues beyond working routines [Table 4, Quote 14].

Our data indicate that the behaviour of the GPs had to some extent an impact on the PrAs’ behaviour. We observed that some GPs took no breaks during consultation hours. This was also addressed by the PrAs in one focus group discussion [Table 4, Quote 15]. In the same practice, a PrA stated that practice staff would often prepare documents during their lunch break. We observed the GP in the same practice taking at least short breaks. In one practice, the lunch breaks were taken outside the practice by both GPs and PrAs.

### Organizing predictable and unpredictable absence of staff

A further dimension of working time organization was the predictable and unpredictable absence of staff. Vacations go along with plannable absence of staff. We found different forms of collective and individual regulations for vacations in the practices observed. In this context, it was of utmost relevance whether one or several GPs worked in a practice. In single practices, the individual vacations of the GP automatically led to collective vacations of the whole practice team as the practice closed completely. In group practices, this was rather the result of a choice made by the practice owners: some chose for simultaneous vacations of all GPs and thus collective vacations and a closed practice. Others chose for individual vacations of the GPs and a practice that remained open with a reduced patient volume. Independent from these regulations, (additional) individual vacations were possible for PrAs across all practices. GPs in all practices delegated individual vacation planning to the practice staff under the condition that practice work would run smoothly and without conflicts [Table 5, Quote 16]. GPs showed willingness to support PrAs in their individual vacation planning, e.g. by adjusting the patient volume during vacation periods [Table 5, Quote 17]. The practice staff described mutual social support when planning individual vacations [Table 5, Quote 18].

In contrast to vacations, sick leaves of GPs or PrAs occur expectedly, but unpredictably. Overall, sick leaves were rather addressed with regards to the PrAs, while sick leaves of GPs were hardly discussed and was observed in one group practice only. A GP attributed this to the traditionally strong work ethics among GPs [Table 5, Quote 19]. Some GPs chose to handle sick leave of staff spontaneously. In these cases, tasks, rosters and patients were re-organized the day a staff member became sick. Either the GPs organized this themselves [Table 5, Quote 20] or delegated the task to the PrAs [Table 5, Quote 21]. Some PrAs explained that an organization of sick leave through the PrAs was beneficial for the organization of work as they had a more complete overview of the tasks at hand and the availability of PrAs [Table 5, Quote 22]. At the same time, a GP of the same practice criticized the described procedure of the PrAs if it was not authorized by the GP. From the GP’s point of view, the ultimate authority over decision-making on work re-organisations belonged to the GP. Consequently, conflict might arise when decisions were made by the PrAs without approval by the GP [Table 3, Quote 4].

Some GPs took preventive measures and thus planned the unpredictable absence of staff. One GP emphasized their responsibility for the planning of the staffing ratio as a precautionary measure to avoid spontaneous shortages due to illness [Table 5, Quote 23]. In a different practice, an on-call roster of the practice staff was drawn up for the entire year by a GP in cooperation with a staff member. Thus, little organizational effort was supposed to be required in case of sick leaves and possible stress due to short-term planning or insufficient staffing was supposed to be prevented [Table 5, Quote 24]. As the quote indicates, such an approach was also the result of a learning process, meaning that systems are constantly reworked and improved. In one of the group practices observed, the mutual substitution of the GPs for each other was contractually regulated. According to the contract, the GPs would cover each other’s absence for 6 weeks and then split the revenues.

## Discussion

### Key roles of GPs and leadership behaviour

The organization of working time is one example for the complexities that arise on a daily basis in GP working environments in Germany. It means planning the unpredictable such as exact daily patient volumes, workload, breaks, sick leave, and vacations. We focussed on this issue for our research about occupation health and safety in GP teams because the organization of working time is a key factor in occupational health. Furthermore, it is particularly challenging as several, potentially conflicting roles of the GP coincide in this process: the GP as healthcare professional, employer, entrepreneur, and leader. Role-specific requirements and responsibilities need to be balanced by the GPs in each practice. As healthcare professionals, accurate and adequate treatment for every single patient includes direct patient contact as well as indirect tasks related to patient care such as documentation. GPs may consider financial aspects (entrepreneur) as well as good access to health care for many patients (healthcare professional) when setting the consultation hours. As leaders, the GPs determine whether appointment scheduling, walk-in consultation hours or a hybrid model will be implemented to organize consultation times. As entrepreneurs, GPs decide e.g. on the spatial design, financial requirements, availability of qualified staff and patient volume to compensate for sick leave and vacation time. GPs as employers are responsible for occupational health and safety, and e.g. define whether break rooms will be available for employees and whether vacations will be taken collectively or individually. As leaders, GPs not only define tasks and responsibilities including which ones to delegate, but also set standards as role models for communication styles and health behaviour. Nowadays, the roles of “leadership”, “management”, and “health professional” are part of the CanMEDS model [5] and its 2015 adaption by the German College of General Practitioners and Family Physicians [6]. Yet this did not apply to the former postgraduate training of most GPs integrated in our study and beyond. As 72,5% of the German GPs are older than 49 years [41], they received their training mostly in the clinical setting and had to learn practice management at a later stage in their professional career.

Some GPs apply a *transactional leadership behaviour* [42], e.g. when they are the ones who organise practice staff either spontaneously or via back-up rosters in case of sick leave. Some GPs applied a *transformational leadership behaviour* [43] when they set an example for taking breaks. GPs have a role model function and their behaviour can guide employees [43]. The results of a longitudinal study confirm the relation between leaders’ handling of their own health and the health of their employees, and that active health-related behaviour by leaders motivates employees to positively develop their own health behaviour [44]. In addition, a positive example for the execution of breaks by the GPs could therefore motivate the PrAs to realize their own breaks in a similar way and reflects a transformational leadership behaviour [45]. Another example of a more transformational leadership behaviour is the delegation of the organization of breaks, vacations or sick leave coverages to their practice staff, if it was grounded in trusting the practice staff to organize details within the overarching aims of the practice. Our data indicate that such practice routines are not necessarily grounded on clearly communicated decision and delegation approaches, but are sometimes rather the result of a habit that worked in daily practice and becomes noticeable in case of miscommunication, disturbances, or disagreements of the GP with the staff’s decision.

### Leadership and practice staff’s psychosocial demands and resources

Different aspects of leadership are in a complex interplay with different demands and resources [46] for both employees and GPs. Predictable working hours as a result of staff-orientation affect the organisation of private life and, as a work-related resource, can improve the work-privacy balance [14], whereas unpredictable working hours as a consequence of poor work organisation can lead to greater work-privacy conflicts and perceived stress [47]. Also, it can result in exhaustion of GPs and practice staff if patient volume-orientation results in high workload and/or the perception that work never finishes [3]. On the other hand, different working time models can be a work-related resource for practice staff. Part-time work models in general practice can improve the balance between private life and work, and may result in better work-privacy balance and higher job satisfaction compared to full-time employees [48]. However, employees in German GP practices are mostly female and thus share the disadvantages of part-time employment with working women in other sectors: short working hours are associated with lower income and increase the risk of poverty, e.g. as single-parent households and lower pension entitlements later on [49]. For employers, a high proportion of part-time workers may affect the functionality of the practice organization [49] and may impair continuity of care.

On the one hand, leadership behaviour which is characterized by more well-communicated delegation, empowerment and/or trust in the staff’s capabilities may foster psychosocial resources as it increases the practice staff’s scope for action. It enables employees to adjust breaks as well as vacation planning more self-determined to their daily rhythms respectively their private lives. On the other hand, rather passive behaviour of a leader regarding relevant working time issues in combination with a practice team less proactive in self-organization may also result in work-related stress. We observed that PrAs sometimes could not organise sufficient breaks during working hours, a finding that was also confirmed in other studies [24]. Sufficient breaks enable recovery and stress compensation and thus have a positive influence on mental health [50]. In contrast, the shortened or absent breaks observed in our study can lead to a permanent workload without sufficient recovery periods and to exhaustion [2]. The Federal Institute for Occupational Safety and Health recommends structurally anchoring the organization of breaks and the establishment of a good “rest break culture” in enterprises [50] which is the responsibility of the employer. Also, passive leadership behaviour of the employer may result in unclear responsibilities and expectations or conflicting instructions. Here, implicit delegation in the sense of laissez-faire leadership behaviour can lead to short-term empowerment [45], but psychosocial demands. More active leadership behaviour can be relieving for the practice staff as it clearly defines areas of responsibility between GPs and staff members beyond what is defined through legal regulations. Clear responsibilities within the practice team are associated with a higher job satisfaction [48]. Furthermore, our data indicate that an active leadership behaviour that compensates the unplannable aspects of working time organization can convey a sense of control and predictability to the practice staff and thus function as a psychosocial resource [3].

Independently from the leadership behaviour of the GP, our results presented positive social relationships in the workplace, both with GPs and practice staff as work-related resources. It has become particularly visible that social support can mitigate psychosocial risks [51], e.g. in the context of predictable working hours and the organization of vacations. Collegial support and the support of leaders are strengthened by positive social relationships and a positive working atmosphere; the resulting mutual relief can be a work-related resource [24]. Furthermore, while a clear division between work and private life was generally observed among practice staff, this was not the case among GPs. GPs reported that they also worked on patient and practice management issues in the evenings, at weekends and at home. On the one hand, this can help to reduce tensions resulting from the multiple roles during consultation hours, on the other hand, this can lead to undefined work ends [3] with a limited spatial and temporal separation from work dissolving boundaries between work and private life [1].

In GP practices, economic aspects can become relevant depending on the structure of the practice. This becomes particularly clear in single practices when the multiple roles of GPs are required simultaneously and overlapping: GPs’ absences due to illness can be associated with loss of income at constant cost and a longer period of incapacity to work can lead to a financial burden [52] some of which can be covered by insurances. Our results indicate that GPs are rarely absent due to illness. This can be linked to results from studies showing that physicians working in general practice or as a private specialist would work even if they were ill [53]. In addition to financial aspects, other factors can promote so-called presentism [54]. It can be particularly difficult for physicians who work alone or in small practices to find a substitute, and long waiting lists and a sense of responsibility towards patients and colleagues could contribute to physicians coming to work sick [53, 55]. Presentism, however, can result in exhaustion and depersonalization and depression [54]. On the other hand, many physicians enjoy being self-employed and report a high job satisfaction as they value this as an important health resource for themselves [25–27].

### Strengths and limitations

Our study is an ethnographic study which consists of observational data, interviews and focus groups discussions to gain deeper understanding of processes and concepts at play in general practices in Germany. To our knowledge, it is the first study of this kind in this setting in Germany. The team-based ethnographic approach enabled the research of work processes and resulted in work-related demands and resources. It became clear that, despite similarities and patterns, each practice is its own unique microcosmos. The findings from the various interviews and observations complemented each other, e.g. by observing some aspects that were not explicitly addressed by the participants neither on site nor in the interviews or focus groups. For example, the organization of the breaks only became clear through the combination of different methods: the availability and utilisation of break rooms were observed, whereas the significance of the break rooms for GPs was identified in individual interviews. The realisation of breaks by GPs as role models was partly recorded through a combination of observations and focus group discussions. The result is an insight into how differently break cultures are established in GP practices and that intention (short breaks happen and are handled flexible) and reality (hardly any short breaks are made) sometimes differ. There are, however, some unintended “blanks” [56] in our results: it is possible that relevant aspects were consciously not addressed by the GPs and PrAs or that given the abundance of received information, the researchers may not have asked for specific procedures, even if there were opportunities to do so. In addition, certain aspects might not have been included in the data as they were not relevant in the observed practices at the time of data collection. Therefore, we have no results regarding reintegration of (long-term) sick practice staff or the organization of working time during pregnancy and maternity protection, or parental leave. Furthermore, data were collected in early 2018, and legal regulations have changed in May 2019 which increased the number of legally required consultation hours, in turn affecting the organization of consultation hours (walk-in vs. appointment system) [57]. It will need to be seen whether and how this might change working time organization in GP practices. Our data indicate that leadership behaviours are independent from legal regulations and the law will rather affect GPs as entrepreneurs.

We cannot provide quantifiable details and results as this does not fit the research design and the aim of our study. The number of the five practices for the ethnographic study was appropriate to achieve the research target, which was to capture diverse work processes in the setting of GP practices and the resulting psychosocial factors. The sample size complies with other ethnographic research designs [31]. All observed practices were part of a university teaching practice network. Thus, a successful motivation and an open-minded attitude of the practice teams towards the subject of the study was noted, e.g. as access to the GP practices went very well. Nevertheless, the data represent a variety of different work processes and work-related resources as well as demands and critical voices. The credibility and transferability of the results was validated by an independent research support group with additional GPs and PrAs [58]. An intervention has been developed in the subsequent module of the joint project [33].

## Conclusions

In this study we highlighted that GPs fulfil several roles and different leadership tasks regarding the organization of working time. GPs can face tension or perceive psychological stress in their multiple roles when considering their responsibility for the health of their employees from different perspectives. As GPs practices are an example of micro and small enterprises, this field of tension could be relevant for entrepreneurs and leaders of enterprises of other occupational fields as well.

GPs seem to have varying levels of awareness of the implications of different leadership styles and their leadership responsibility in the organization of working hours, different possibilities of implementation and the resulting consequences for themselves and their employees. This is in line with a study on Norwegian GPs which found that GPs have uncertainties in carrying out the leadership role due to insufficient education and training for the leadership role [59]. This might also result from models that are rather oriented towards the clinical than the ambulant setting. GPs could be supported by greater integration of leadership tasks and skills during medical studies or by offering special leadership training courses [60]. This would enable GPs to meet the diverse demands of their different roles in a more health-oriented way. However, 35,2% of the GPs in Germany are 60 years or older [41], which might affect their interest in investing in training for the remaining years of their working life.

Especially with regard to working time organization, GPs do not seem to be an exception. The majority of employers in German small and medium sized enterprises (SMEs) is not familiar with the Working Time Act [9] and when asked who is legally responsible for occupational health and safety, only 60.7% of the employers surveyed stated that they are the ones solely responsible [61]. Sczesny et al. [61] conclude that personal responsibility for occupational health and safety in the workplace is thus not part of the self-evident basic knowledge of employers in SMEs. The low importance of occupational health and safety is explained by the large number of major and sometimes conflicting interests and objectives [61]. In principle, leadership training seems advisable as part of the training of future leaders of micro-enterprises, for example to promote self-reflection and measures that are tailored to the uniqueness of each company. In this process, the companies´ goals and leadership responsibilities can be reflected upon, taking into account the working conditions and consequences for the personnel.

## Supplementary Information

**Additional file 1.**

**Additional file 2.**

## Data Availability

Not applicable.

## Abbreviations

GP: General practitioner
PrA: Practice assistant
SME: Small and medium-sized enterprises
