# Supplementary material for: Psychosocial demands and resources for working time organization in GP practices. Results from a team-based ethnographic study in Germany
Source: J Occup Med Toxicol. 2021 Oct 18;16:47. doi: 10.1186/s12995-021-00336-w (PMC8522246; doi:10.1186/s12995-021-00336-w)
Supplement: Supplementary file 2 — Additional file 2. [file 12995_2021_336_MOESM2_ESM.docx]

**Supplementary material 2: IMPROVE*job-*Consortium**

**Current institutions and members:**

*Institute of Occupational and Social Medicine and Health Services Research, University Hospital Tuebingen, Germany:* MA Rieger, E Rind, A Siegel, A Wagner, S Burgess

*Department of Psychosomatic Medicine and Psychotherapy, Medical University Hospital Tuebingen, Germany:* F Junne, T Seifried-Dübon, F Stuber, A Herrmann-Werner, S Zipfel

*Institute of General Practice and Family Medicine, University Hospital Bonn, Germany:* B Weltermann, S Kasten, K Linden, L Degen, J Göbel, M Schmidt

*Operations Research, Ruhr-University Bochum, Germany:* B Werners, M Grot

*Institute for Medical Informatics, Biometry and Epidemiology & Center for Clinical Studies, University of Duisburg-Essen, Germany:* K-H Jöckel, C Pieper, V Schröder, A-L Eilerts, M Brinkmann

**Former institutions and members:**

*Institute for General Medicine, University Hospital Essen, Germany:* C Kersting

*Institute of Occupational and Social Medicine and Health Services Research, University Hospital Tuebingen, Germany:* S Hartmann (née Emerich), M Hippler, E Tsarouha

*Institute of General Practice and Family Medicine, University Hospital Bonn, Germany:* A Dreher

*Institute for Medical Informatics, Biometry and Epidemiology & Centre for Clinical Trials, University of Duisburg-Essen, Germany:* C Ose, J-M Bois

*Operations Research, Ruhr-University Bochum, Germany:* L Imhoff (née Koppka), J Block
